# Incorporating dynamic flight network in SEIR to model mobility between populations 

**DOI:** 10.1007/s41109-021-00378-3

**Published:** 2021-06-10

**Authors:** Xiaoye Ding, Shenyang Huang, Abby Leung, Reihaneh Rabbany

**Affiliations:** 1 School of Computer Science, McGill University, Montreal, Canada; 2 Mila, Quebec Artificial Intelligence Institute, Montreal, Canada

**Keywords:** Epidemiological modelling, Dynamic network, Network science

## Abstract

Current efforts of modelling COVID-19 are often based on the standard compartmental models such as SEIR and their variations. As pre-symptomatic and asymptomatic cases can spread the disease between populations through travel, it is important to incorporate mobility between populations into the epidemiological modelling. In this work, we propose to modify the commonly-used SEIR model to account for the dynamic flight network, by estimating the imported cases based on the air traffic volume and the test positive rate. We conduct a case study based on data found in Canada to demonstrate how this modification, called Flight-SEIR, can potentially enable (1) early detection of outbreaks due to imported pre-symptomatic and asymptomatic cases, (2) more accurate estimation of the reproduction number and (3) evaluation of the impact of travel restrictions and the implications of lifting these measures. The proposed Flight-SEIR is essential in navigating through this pandemic and the next ones, given how interconnected our world has become.

## Introduction

The coronavirus disease 2019 (COVID-19) pandemic spread rapidly around the world and has affected countless lives. As of March 14, 2021, there have been 119,220,681 confirmed cases and 2, 642,826 deaths globally (WHO 2020a). Of all places, but the origin, the start of the spread is related to travel. For example, in Canada, the first travel-related case was identified in January 2020 and by April 2020, community transmission was present in almost all provinces (Ogden et al. 2020). To better understand and effectively model a global pandemic such as COVID-19, it is important to consider the following questions: (1) *how to model the spread of a disease from one community to another*, (2) *how to estimate the likelihood of an outbreak caused by imported cases* and (3) *how to measure the effectiveness of travel bans and evaluate the impact of lifting those restrictions*. In this work, we aim to answer the above questions by incorporating dynamic flight network into epidemiological modelling.

**Fig. 1:**
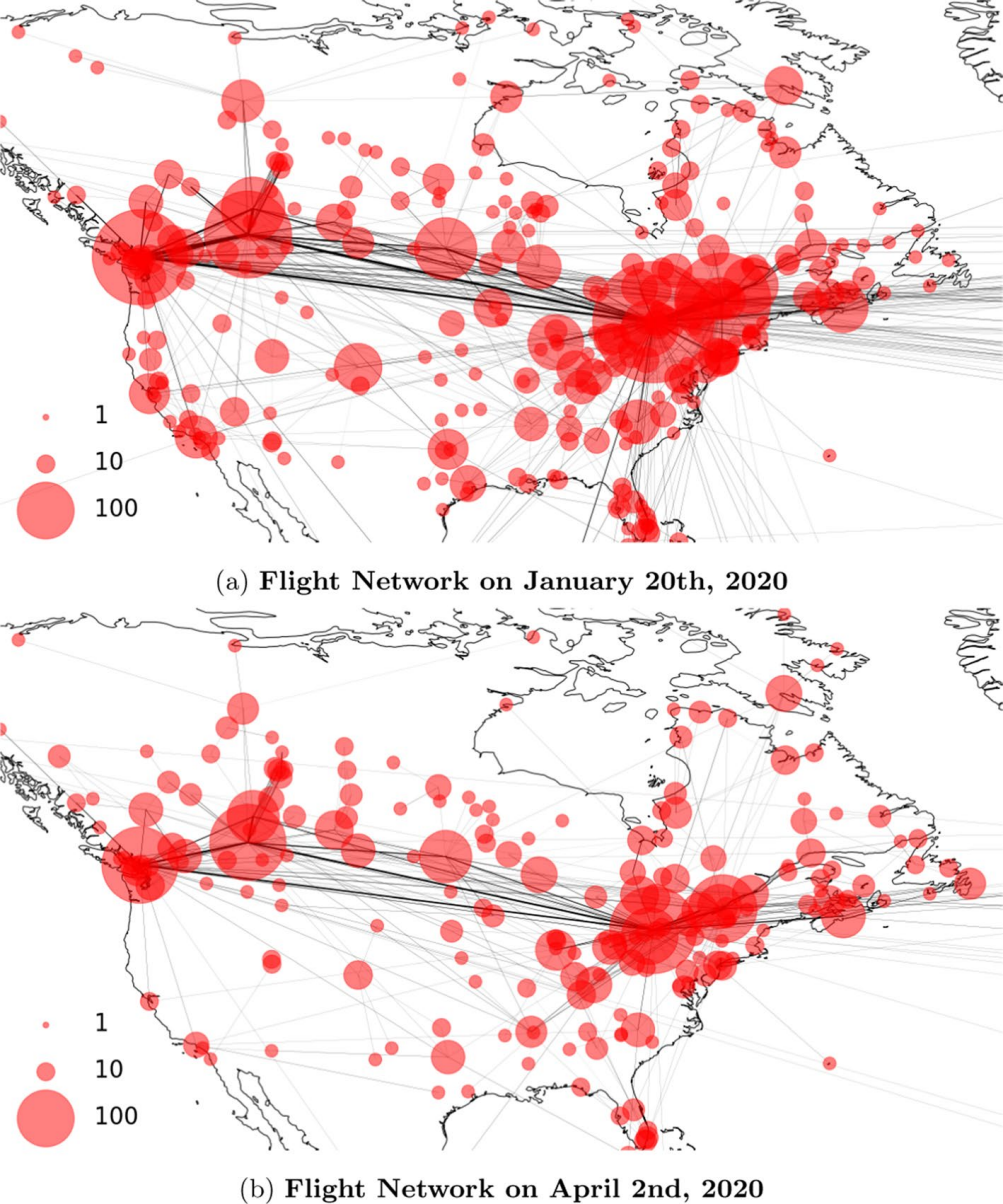
Flight network before and after imposing travel restrictions. Note that only flights with an endpoint in Canada have been considered

Most models view each population as a closed system, isolated from the rest of the world. However, this assumption is rarely true in practice. For example, in Canada, even with the travel restrictions in place, there are still a considerable amount of flights going in and out of the country. As shown in Fig. 1, while there was a significant amount of reduction in daily flights to and from Canada, much air traffic remained well after the travel restrictions were put in place, i.e. March 16th with countries excluding the United States(US) and March 20th with the US. Here, each node represents one of the airports in the US and Canada. Both the size of the node and the thickness of the edge are in proportion to the volume of flights. Note that we focus on flights data arriving at or departed from Canada, which is the population under study for this work. The node size of US airports only reflect their traffic volumes related to Canada.

To account for mobility between populations, we propose Flight-SEIR, a modification of the standard susceptible, exposed, infected and recovered (SEIR) model to consider travellers between the target population and the rest of the world. We show how Flight-SEIR is able to raise alarms of upcoming outbreaks even when there is no community transmission. We believe that Flight-SEIR is crucial for alerting public agencies ahead of an outbreak similar to COVID-19. Moreover, we show that Flight-SEIR is able to fit the real data more closely and provide a better estimation of how contagious the disease is when compared to the standard SEIR model which either underestimates or overestimates the size of infectious population by assuming a closed population. In the context of COVID-19, there is an immense political and economical pressure for the governments to reopen the borders and resume flights. Flight-SEIR can inform policy makers by predicting the epidemic curves with projected flight data therefore performing risk estimation of lifting air travel restrictions.

## Related works

Many recent works model COVID-19 with variants of the classical susceptible, infected and recovered (SIR) and the susceptible, exposed, infected and recovered (SEIR) model (Katul et al. 2020; Ogden et al. 2020; Tuite et al. 2020; Linka et al. 2020). The focus of these studies is often placed on the estimation of the basic reproduction number $R0$ (Katul et al. 2020), the average number of new infections an infected individual causes in a fully susceptible population (Barabási 2013).

One key challenge of modelling COVID-19 is the existence of asymptomatic and pre-symptomatic transmission (Wei et al. 2020; Vetter et al. 2020). These pre-symptomatic and asymptomatic carriers of COVID-19 can travel to a foreign country and initiate the spread of COVID-19 which eventually leads to an outbreak. Motivated by this, some studies consider the travel between populations. For example, Bogoch et al. (2020) estimated the risk of an outbreak by presenting the Infectious Disease Vulnerability Index (IDVI) for international destinations receiving the highest number of passengers from major cities in China. Kraemer et al. (2020) and Linka et al. (2020) studied the correlation between human mobility and the spread of the disease. These models are, however, not epidemiological models and cannot project the spread of disease based on travel patterns.

Lin et al. (2020) introduced a step-wise emigration rate to model the before and after of Wuhan lock down. Yang et al. (2020) considered a dynamic population with inflow and outflow of susceptible and exposed individuals between different provinces in China. The size of inflow and outflow depends on the migration index, defined based on the number of inbound and outbound events by rail, air and road traffic. Yamana et al. (2020) assessed the impact of reopening states in the US by considering the daily work movements and random movements between counties. However, these models only consider disease spread within the same population. In comparison, our method also incorporates international flights to effectively understand how COVID-19 spreads globally.

Some models do account for the global spread of COVID-19. Kucharski et al. (2020) studied domestic cases within Wuhan and international cases that originated from Wuhan. In particular, they modelled how individuals that are exposed to COVID-19 in Wuhan can spread the disease to other countries. They assumed that the probability of cases being exported from Wuhan depended on the number of cases in Wuhan, the number of outbound travellers, the relative connectivity of different countries and the relative probability of reporting a case in each country. Wu et al. (2020) inferred $R0$ and the outbreak size by estimating the number of cases exported from Wuhan. They also forecasted the spread of the disease within and outside Wuhan by accounting for mobility and transmissibility reduction. Chinazzi et al. (2020) applied the Global Epidemic and Mobility Model to simulate the effect of travel ban in China. International mobility is set to reduce by 40% and 90% after the travel ban while domestic mobility is derived from mobility indices. These models either assume that the global travel behavior remains the same throughout the pandemic or used a step-wise function to model mobility patterns before and after international travel restrictions. In contrast, Flight-SEIR incorporates dynamic flight network which models more granular changes in the air traffic and provides the flexibility of plugging in projected flight network to simulate different levels of travel restrictions. In addition, we are not aware of any work that studies how resuming flights will affect the spread of COVID-19, which Flight-SEIR provides.

While travel-related Non-Pharmaceutical Interventions (NPIs) played a significant role in confining COVID-19, some studies focused on other NPIs such as social distancing and quarantine (Tuite et al. 2020; Ogden et al. 2020; Ferguson et al. 2020; Block et al. 2020; Reich et al. 2020). For instance, Ogden et al. (2020) described the predictive modelling efforts for COVID-19 within the Public Health Agency of Canada. They modelled the effects of physical distancing by reducing daily per capita contact rates. A separate agent model is used to simulate the effects of closing schools, workplaces and other public places. Ferguson et al. (2020) employed an individual-based simulation model to evaluate the impact of NPIs, such as quarantine, social distancing and school closure. Reich et al. (2020) compared the impact of testing, contact tracing, quarantine and social distancing with a network-based epidemic model.

## Flight-SEIR

**Fig. 2:**
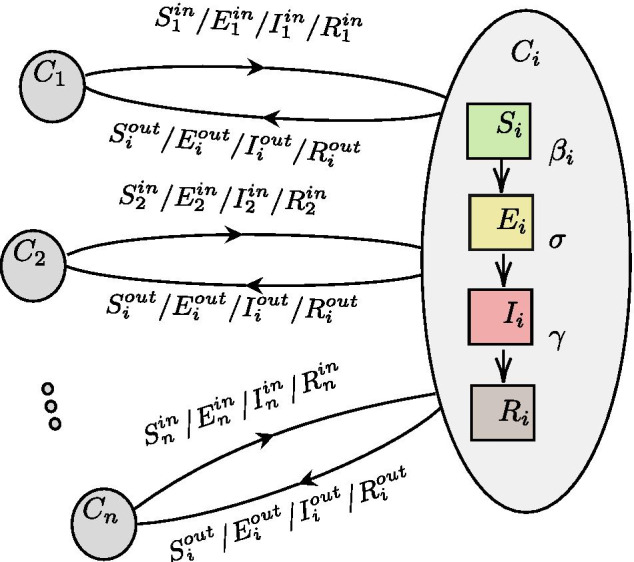
Demographic and epidemic dynamics of Flight-SEIR. The figure shows the movements of exposed individuals $E_{i}^{in}$ and $E_{i}^{out}$ between the populations. Each population $C_i$ maintains its own epidemic states $S_i$, $E_i$, $I_i$, and $R_i$. An exposed individual can either come from other populations or be infected by an infectious individual within the same population. More details on the notations can be found in Table 1

We modified the differential equations of a standard SEIR model to include demographics dynamics derived from the flight network. This modified SEIR model is called Flight-SEIR. More specifically, we consider an open population setting where people are free to travel in and out of the population. Figure 2 illustrates the epidemic and demographic dynamics modelled by Flight-SEIR, and Table 1 summarizes the notations used in Flight-SEIR. More formally the modified equations are as follows:

1 $$\begin{aligned}&\frac{dS_{i}}{dt} = - \frac{\beta _i S_{i}I_{i}}{N_{i}} + {\sum _{i} S_{ji} - \sum _{i} S_{ij}} \end{aligned}$$

2 $$\begin{aligned}&\frac{dE_{i}}{dt} = \frac{\beta _i S_{i}I_{i}}{N_{i}} - \sigma E_{i} + {\sum _{i} E_{ji} - \sum _{i} E_{ij}}\end{aligned}$$

3 $$\begin{aligned}&\frac{dI_{i}}{dt} = \sigma E_{i} - \gamma I_{i} + {\sum _{i} I_{ji} - \sum _{i} I_{ij}}\end{aligned}$$

4 $$\begin{aligned}&\frac{dR_{i}}{dt} = \gamma I_{i} + {\sum _{i} R_{ji} - \sum _{i} R_{ij}} \end{aligned}$$

where $S_{i}$, $E_{i}$, $I_{i}$, $R_{i}$ represent the size of susceptible, exposed, infectious and recovered population in target population *i*. The incubation rate $\sigma$ is set to be $\frac{1}{C}$ where C is the mean latent period of the disease while the recovery rate $\gamma$ is set to be $\frac{1}{D}$ where D is the mean infectious period. Like the standard SEIR, we assume the latent period and infectious period to be exponentially distributed. The transmission rate $\beta _i$ encapsulates both the transmission rate of the disease and the contact rate of population *i*. We define the population reproduction number $R0_{i}$ as the average number of infections caused by the infectious individuals of population *i*, $I_{i}$. We compute it as $\frac{\beta _i}{\gamma }$. $S_{ij}$, $E_{ij}$, $I_{ij}$, and $R_{ij}$ are the number of susceptible, exposed, infected and recovered individuals travelling from population *i* to population *j*. $S_{ji}$, $E_{ji}$, $I_{ji}$, and $R_{ji}$ are the number of individuals travelling from population *j* to population *i*. In the interest of studying the properties of Flight-SEIR especially during the early time of the pandemic, we make a few assumptions which simplify the modelling without compromising the key characteristics of the model.

1. We assume that individuals who are in the infected compartment, *I*, would be denied boarding flights and therefore cannot travel between populations. Note that both exposed and infected individuals would have received a positive result if they are tested. The key distinction between these two compartments is that infected individuals develop mild to severe symptoms and are easily identifiable. Therefore, we set the number of infected travellers $I_{ij}$ and $I_{ji}$ to 0.
2. Given that the underlying SEIR model does not have a compartment for asymptomatic infected individuals, we ignore them in this work. However the principle of including population flow terms can be extended to more complex compartmental models such as the one used in this work (Ogden et al. 2020).
3. We assume that the number of recovered travellers are negligible during the early stage of the pandemic and their presence will likely not to affect the disease transmission. Therefore, we set the number of recovered travellers $R_{ij}$ and $R_{ji}$ to 0.
4. As the net flow of exposed individuals is insignificant compared to the total population $N_i$, we kept $N_i$ unchanged throughout our simulations.

**Table 1:** Parameters for Flight-SEIR

Parameter	Description	Value	Type
$S_i$	# susceptible individuals at node *i*		
$E_i$	# exposed individuals at node *i*		
$I_i$	# infected individuals at node *i*		
$R_i$	# recovered individuals at node *i*		
$S_{ij}$	# susceptible individuals travelling from node *i* to node *j*		
$E_{ij}$	# exposed individuals travelling from node *i* to node *j*		
$I_{ij}$	# infected individuals travelling from node *i* to node *j*		
$R_{ij}$	# recovered individuals travelling from node *i* to node *j*		
$N_i$	Total population at node *i*		Constant
*C*	Mean latent period of the disease	5	Constant
$\sigma$	Incubation rate $E_i \rightarrow I_i$	$\frac{1}{C}$	Constant
*D*	Mean infectious period	14	Constant
$\gamma$	Recovery rate $I_i \rightarrow R_i$	$\frac{1}{D}$	Constant
$\beta _{i}$	Transmission rate $S_i \rightarrow E_i$ for node *i*		Fitted
$R0_{i}$	The population reproduction number	$\frac{\beta _{i}}{\gamma }$	Fitted
$Passengers_{ij}$	# passengers travelling from node *i* to *j*		Estimated
$Passengers_{ji}$	# passengers travelling from node *j* to *i*		Estimated
$P_i$	Test positive rate of node *i*		Estimated
$\eta$	% of exposed individuals over all infected individuals		Estimated
$F_{ij}$	# flights from node *i* to node *j*		Estimated
*CAP*	Estimated average flight capacity		Estimated
*LF*	Load factor: onboard passengers to available seats ratio		Estimated
$\tau$	% of projected air traffic over pre-pandemic air traffic		Variable

Type of the parameter can be constant, variable, fitted or estimated. Constant parameters are set based on WHO’s Report of the WHO-China Joint Mission on Coronavirus Disease 2019 (2020b) and Government of Canada (2020b)

With the assumptions stated above, we can simplify the update rule to the following:

5 $$\begin{aligned}&\frac{dS_{i}}{dt} = - \frac{\beta _i S_{i}I_{i}}{N_{i}} + {\sum _{i} S_{ji} - \sum _{i} S_{ij}} \end{aligned}$$

6 $$\begin{aligned}&\frac{dE_{i}}{dt} = \frac{\beta _i S_{i}I_{i}}{N_{i}} - \sigma E_{i} + {\sum _{i} E_{ji} - \sum _{i} E_{ij}} \end{aligned}$$

7 $$\begin{aligned}&\frac{dI_{i}}{dt} = \sigma E_{i} - \gamma I_{i} \end{aligned}$$

8 $$\begin{aligned}&\frac{dR_{i}}{dt} = \gamma I_{i} \end{aligned}$$

Next, we describe how to estimate the size of susceptible and exposed travellers, $S_{ij}$, $S_{ji}$, $E_{ij}$, and $E_{ji}$, from the flight network.

9 $$\begin{aligned} E_{ij}&= Passengers_{ij} \times P_{i} \times \eta \end{aligned}$$

10 $$\begin{aligned} E_{ji}&= Passengers_{ji} \times P_{j} \times \eta \end{aligned}$$

11 $$\begin{aligned} S_{ij}&= Passengers_{ij} \times (1 - P_{i}) \end{aligned}$$

12 $$\begin{aligned} S_{ji}&= Passengers_{ji} \times (1 - P_{j}) \end{aligned}$$

where $P_i$ denotes the test positive rate of population *i*, measured in the number of confirmed cases per thousand tests. $\eta$ denotes the percentage of exposed individuals over all individuals who carry the disease, including both the exposed and the infected. $Passengers_{ij}$ and $Passengers_{ji}$ estimate the total number of passenger between population *i* and *j* and are estimated as:

13 $$\begin{aligned} Passengers_{ij}&= F_{ij} \times CAP \times LF\end{aligned}$$

14 $$\begin{aligned} Passengers_{ji}&= F_{ji} \times CAP \times LF \end{aligned}$$

Here, $F_{ij}$ and $F_{ji}$ are the number of outbound/inbound flights from/to population *i* to/from population *j*. *CAP* gives an estimate for the average flight capacity. *LF* proxies the passenger load factor (onboard passengers to available seats ratio) and offers the flexibility of modelling the reduction in passengers per flight due to the pandemic (Airlines for America 2020; Int. Trade Administration 2020).

Why do we estimate the number of exposed individuals going in/out of population *i*, $E_{ji}$/$E_{ij}$ based on the test positive rate *P*? Each population has different testing capacities and strategies. The number of confirmed cases alone does not necessarily reflect the actual number of infected individuals in each population (Krantz and Rao 2020). Test positive rate *P*, on the other hand, gives us a rough idea how many infected individuals we would find if we were to test everyone on the plane. We acknowledge that testing may be limited to symptomatic cases during the early stage and using the test positive rate can lead to an overestimation of infected individuals. However, compared to using the confirmed cases, which underestimate the severity of the situation, we choose to be cautious and work with the possible overestimation. The product of *Passengers* and test positive rate *P* only tells us how many infectious individuals would have come in or out of the population, assuming they have not been prevented from travelling. To derive the number of exposed individuals, we further multiply this with $\eta$, the percentage of exposed individuals over all individuals who would have been tested positive, which include the exposed and the infectious. The same $\eta$ is used across populations. The size of susceptible individuals $S_{ij}$ and $S_{ji}$, on the other hand, is computed as the the number passengers who would NOT have received a positive result if they are tested.

## Estimating mobility between populations from real time data

While Flight-SEIR can be used for modelling the spread of disease over multiple populations, in this paper *we focus on one target population,* *Canada*, to provide more straightforward comparison with the standard SEIR model, as well as to make collecting and estimating relevant data more manageable.

We began our study few months after the pandemic started. Unlike accessing the real-time flight network, *collecting historic flights and estimating population movements is a challenging task*. In this work, we made a few assumptions and we tried our best to validate our estimations against external sources. We believe that Flight-SEIR can be highly informative for decision making at public agencies which have access to more detailed and accurate flights data. We haven’t been able to secure access to such data. Therefore, our experiments represent our best attempt at modelling COVID-19 and serve as a proof of concept for the potentials of incorporating the flight network.

To estimate $F_{ij}$ and $F_{ji}$, we collected historic flights data dating back to Jan, 2020 (Flightradar24 2020). To correct for possible missing flights, we scaled the data so that our account of monthly flights matches the domestic and international itinerant movements reported by Statistics Canada (2020). Based on the flights data, we constructed one snapshot of flight network per day. The nodes are the airports and the edges represent flight connections. They are directed and weighted by the number of flights on that day. As an example, Fig. 1a, b shows the comparison between the flight network on January 20, 2020 and April 2, 2020. The size of the bubble is proportional to the air traffic at the airport. The width of the edge is in proportion to the number of flights between two airports. We observe that there are far fewer flights on April 2 compared to January 20. This can be partially attributed to the travel ban put in place in March (ACAPS: ACAPS COVID-19 2020). It can also be sentiment-related i.e. people are afraid to travel due to the pandemic. To some extent, this dynamic flight network reflects the impact of travel restrictions.

We then further map the airports to countries or provinces and aggregate the number of flights which serve as the input to Flight-SEIR. US passengers, flights and load factor data (U.S.D of Transportation 2020) is used as a proxy to the flight capacity and the load factor since this information is not available for Canada. We compute the average flight capacity as:

15 $$\begin{aligned} CAP = \frac{Passengers}{Flights \times Load Factor} \end{aligned}$$

**Fig. 3:**
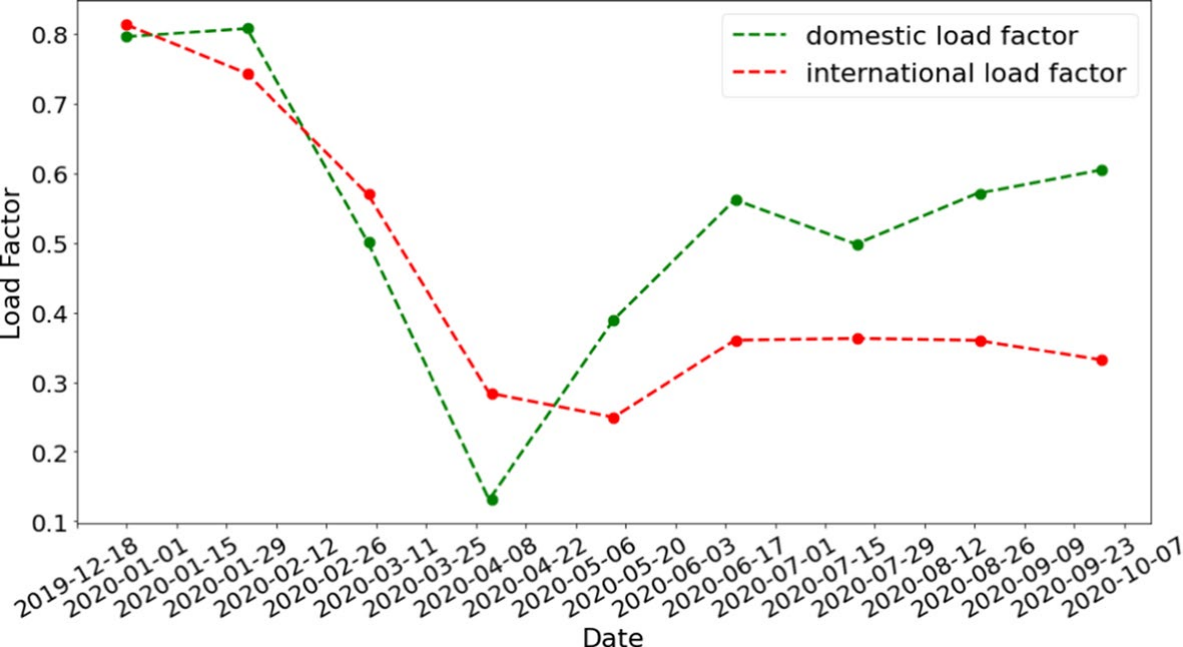
Ratio of passengers to available seats (LF) in flights interpolated based on the available data from U.S.D of Transportation (2020)

CAP is a constant estimated from 2019’s data. More specifically, the total passengers, total flights, and average load factor for domestic flights in 2019 are 811, 547,759 and 8,596,712 and 85.10%. Those for international flights in 2019 are 241,436,079 and 1,623,593 and 82.72%. As a result, *CAP* is estimated to be 111 for domestic flights and 180 for international flights. Figure 3 shows the interpolated load factor *LF*. We can see that both domestic and international load factor have dropped significantly since the start of the year.

**Fig. 4:**
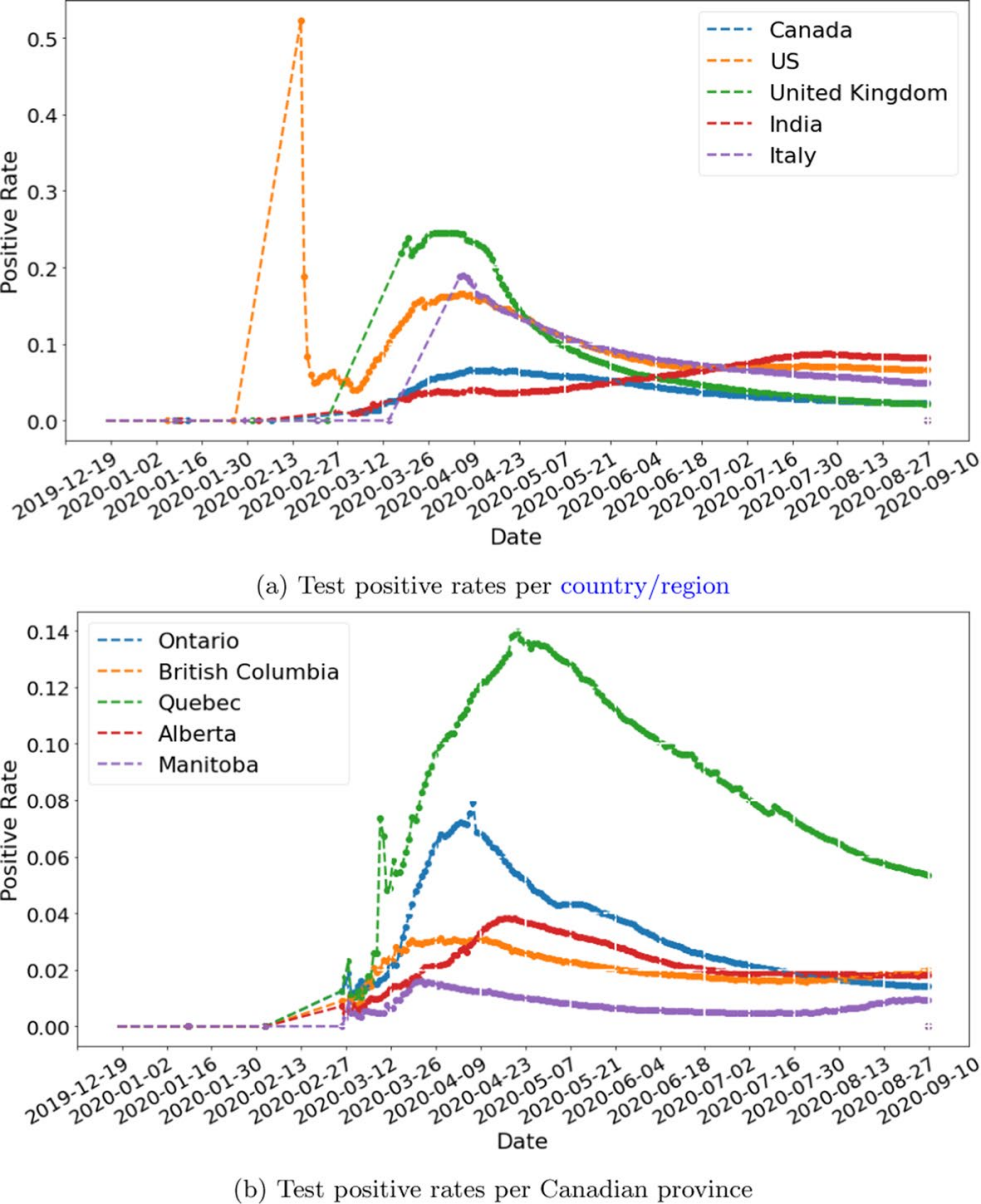
Test positive rates, interpolated based on data from Roser et al. (2020) and Government of Canada (2020a)

**Fig. 5:**
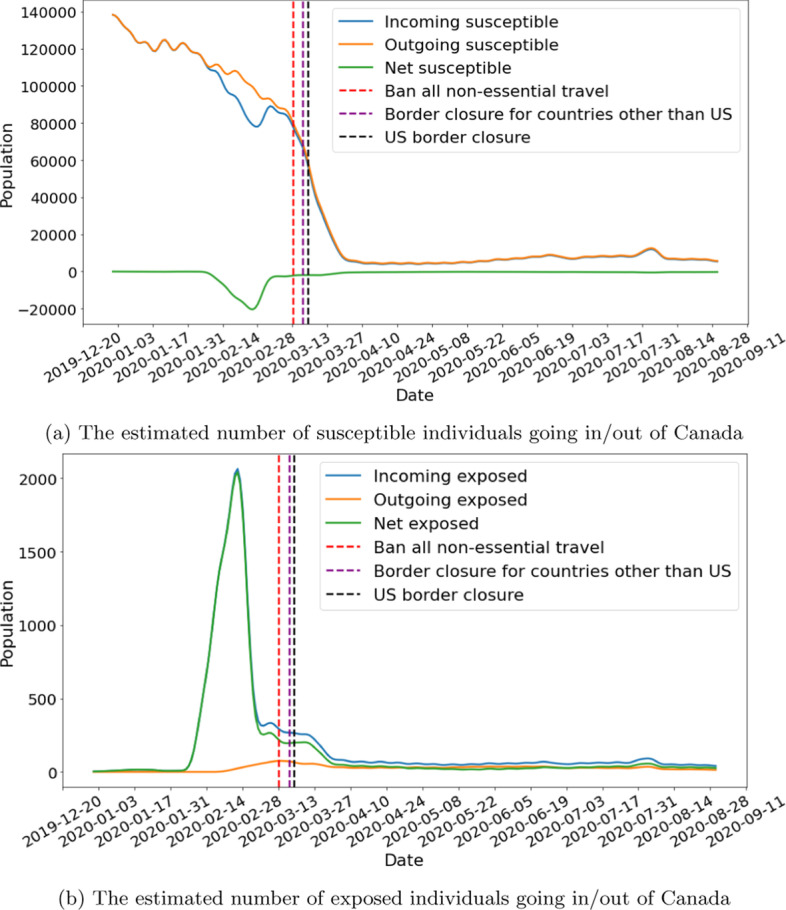
The estimated number of susceptible and exposed individuals going in and out of Canada, before and after travel restrictions

**Fig. 6:**
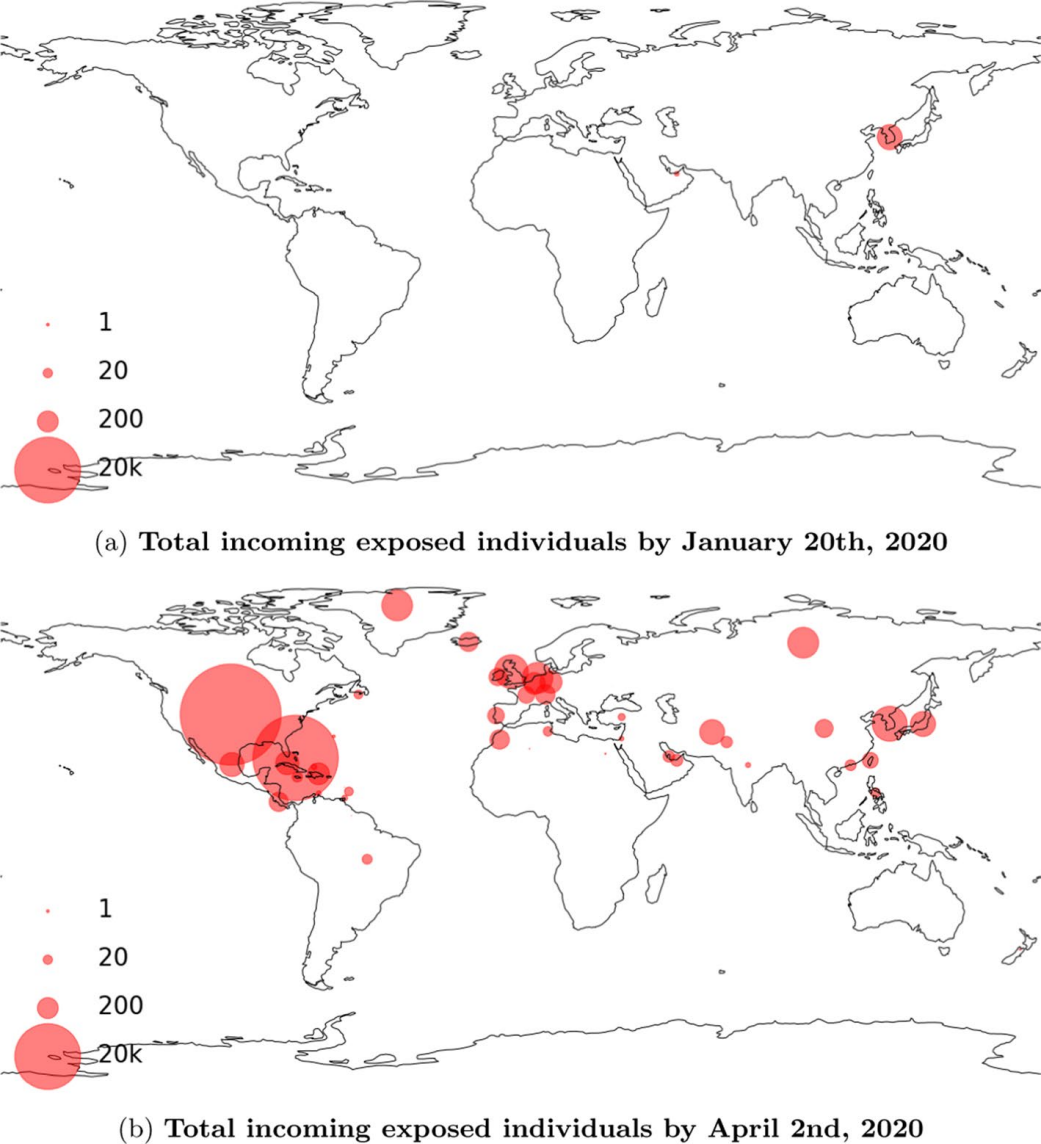
Total incoming exposed individuals to Canada aggregated per country/region. Note that the circle sizes are plotted in log scale

We obtain country/region-level test positive rates from public dataset (Roser et al. 2020) published by Oxford Martin School. For provinces and territories within Canada, we use data published by the Government of Canada (2020a). Figure 4 shows the interpolated test positive rates for selected countries and provinces. $\eta$ is set to 10% to roughly agree with the tracked travel-related (imported) cases reported by Berry et al. (2020). Figure 5 shows the estimated movements of susceptible and exposed people to and from Canada. We observe a spike of incoming exposed individuals in February. The estimation drops after the various travel-related NPIs were put in place. The number of outgoing exposed individuals are low because Canada has a comparatively low test positive rate when compared to the rest of the world. Figure 6 shows our estimation of total incoming exposed individuals to Canada by January 20 and April 2.

*Estimating unrestricted flight volume* We assume the January’s data to be representative of the air traffic prior to COVID-19. Therefore, we scaled the air traffic in the following months to match January’s volume to simulate demographic dynamics without travel restrictions in the early pandemic detection simulations. Moreover, we used the same scaling procedure to simulate the air traffic when we lift travel restrictions and used a scaling factor $\tau$ to control the amount of flights we resume, in the corresponding simulations discussed below. Given that travel restrictions affect both the number of flights and the number of passengers on the plane, we also scale the load factor to account for changes in passengers per flight caused by the relaxation of travel restrictions. The same scaling factor $\tau$ is used to scale both air traffic volume and load factor.

## Simulations and discussions

In the following sections, we describe how Flight-SEIR can be applied to (1) predict uncontrolled early time epidemic dynamics, (2) assess the impact of travel restrictions, (3) estimate the population reproduction number $R0_{i}$ and (4) simulate the effect of lifting travel restrictions.

**Fig. 7:**
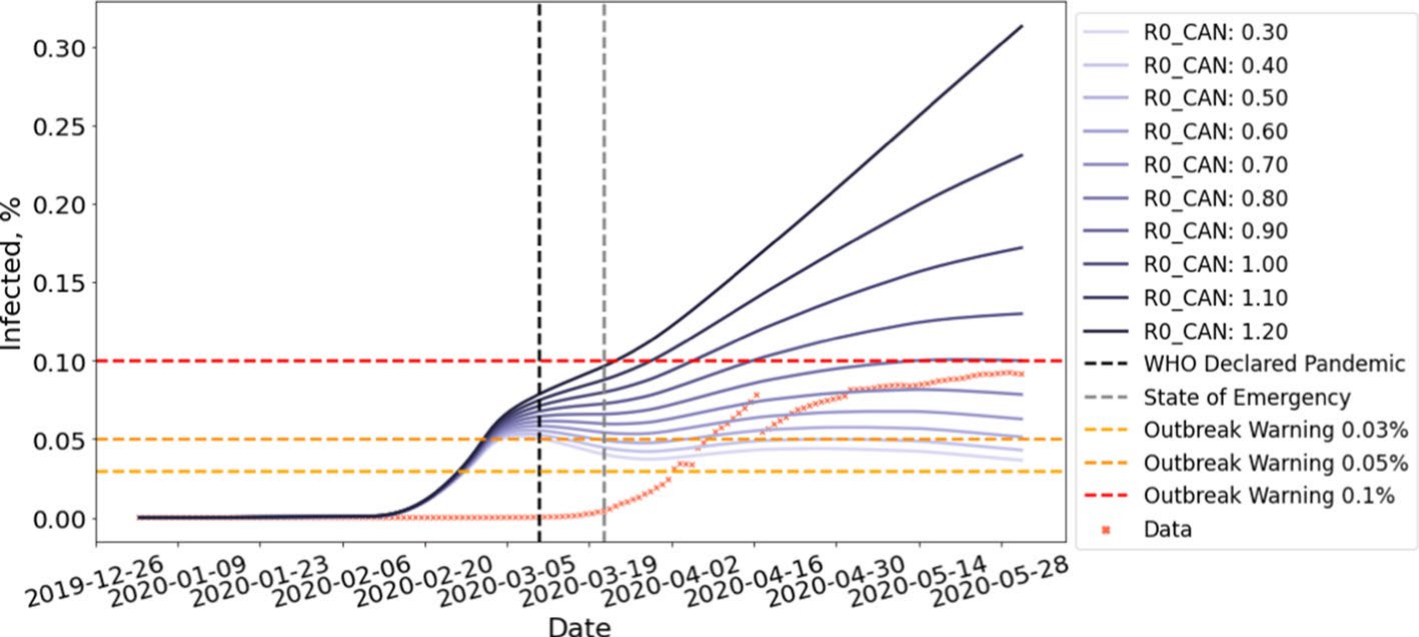
Early time prediction *without* enforced travel restriction. Note that, between April 2–30, a computer error resulted in 1317 missing positive COVID-19 cases in Quebec (CTV News 2020a), causing the gap in the data

**Fig. 8:**
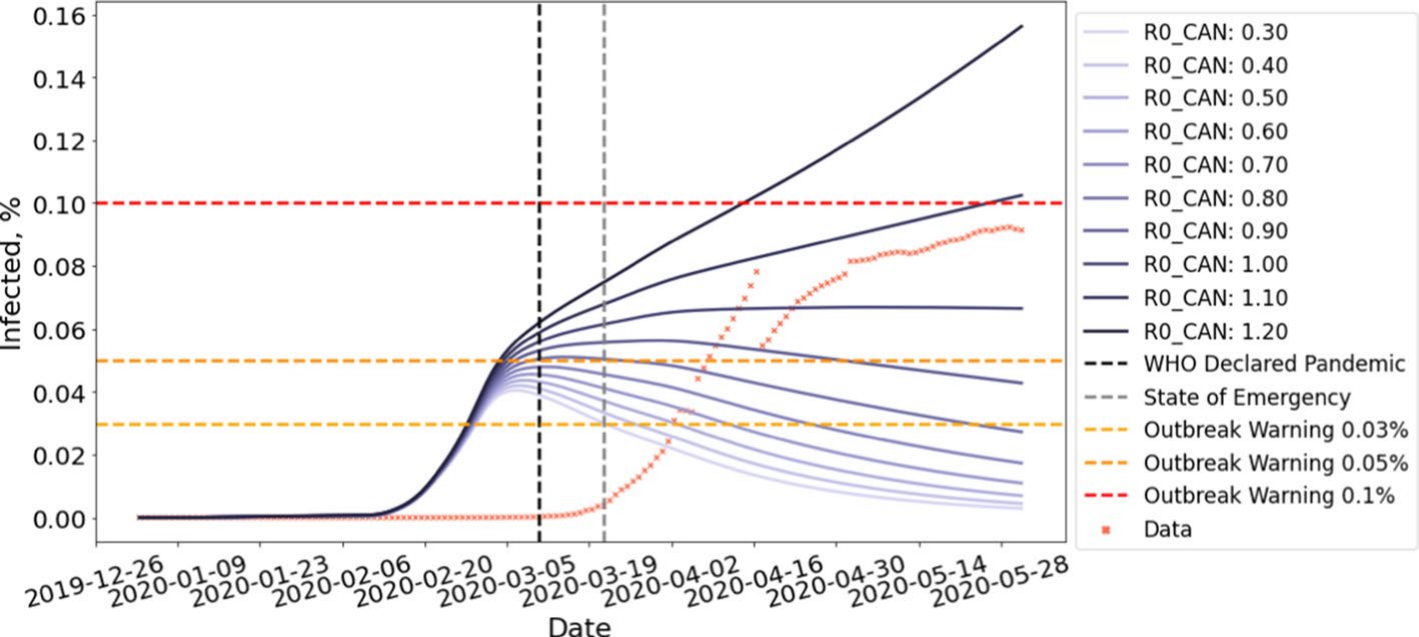
Early time prediction *with* enforced travel restriction

**Fig. 9:**
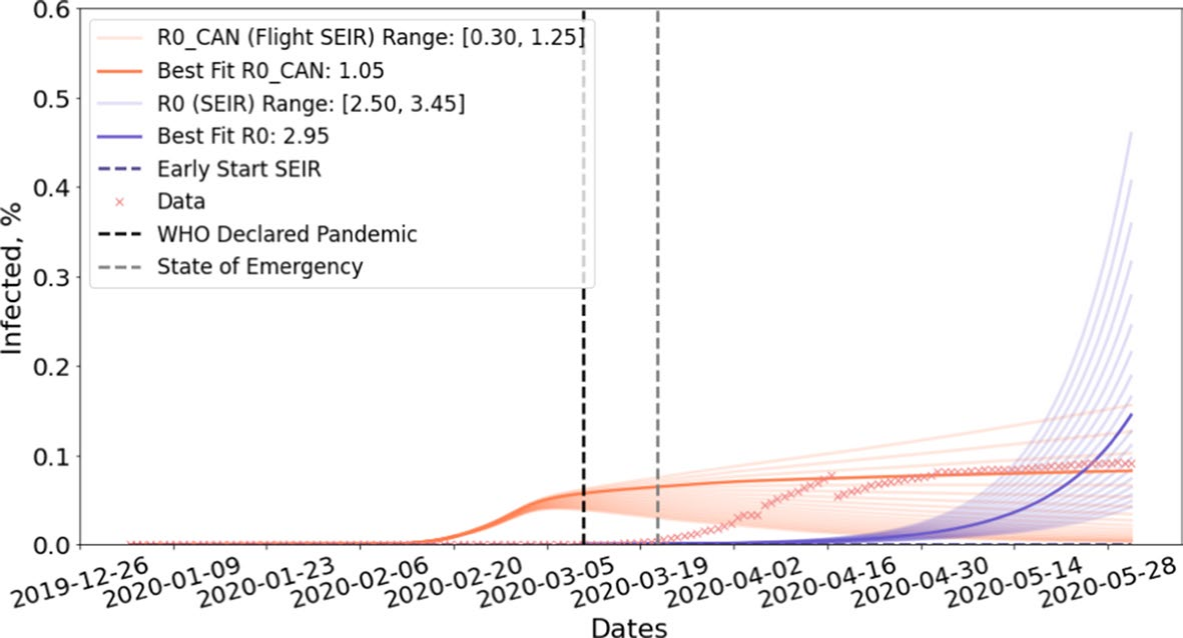
Estimation of $R0_{CAN}$ and $R0$ for Canada. Initially, Flight-SEIR predicts more than the confirmed cases while the standard SEIR model underestimates the number of infectious individuals. Later on, Flight-SEIR fits almost perfectly to the confirmed cases whereas the standard SEIR model starts to overestimate

**Fig. 10:**
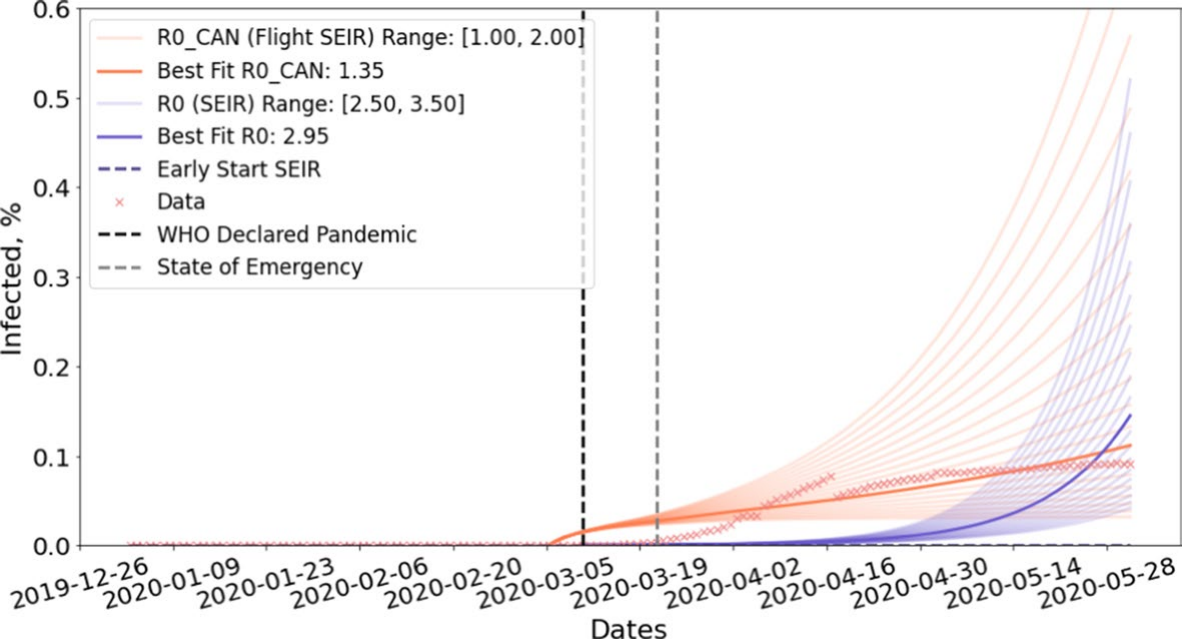
Estimation of $R0_{CAN}$ and $R0$ for Canada given starting conditions. Estimation of $R0_{CAN}$ and $R0$ assuming that the simulation starts on March 6 and 20% of infected cases are reported. The degree of early phase overestimation is alleviated while the fit to later phase remains reasonable

**Fig. 11:**
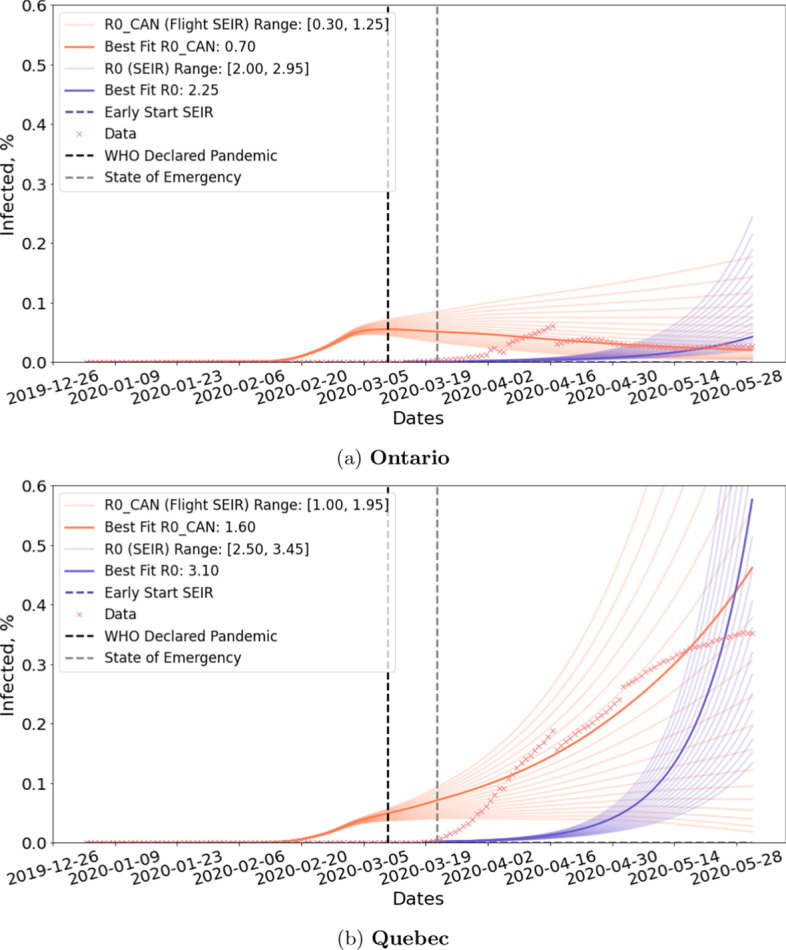
$R0_{CAN}$ Estimation for difference provinces within Canada. Quebec is estimated to have a much higher $R0_{CAN}$ than Ontario. In both cases, Flight-SEIR shows a better fit than the standard SEIR model

**Fig. 12:**
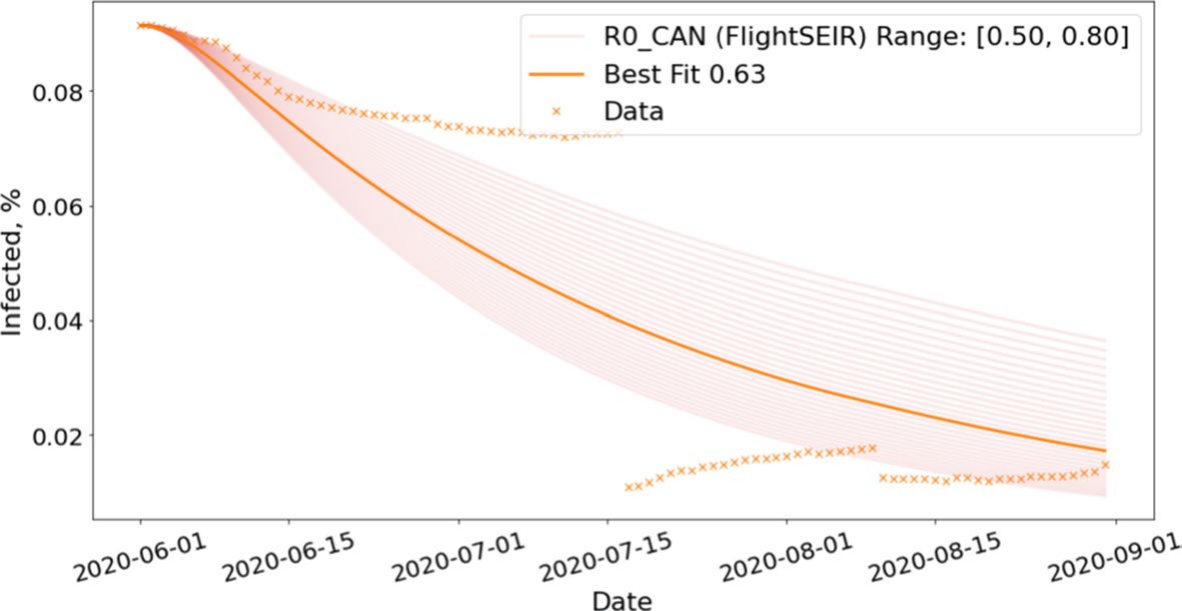
$R0_{CAN}$ estimation for reopening simulation. Flight-SEIR is fitted to confirmed cases from June 1st to August 1st, approximately 2 months before the reopen date. We run grid search in the range of [0.50, 0.80] and find the best fit to be 0.63. Note that, on July 17, Quebec revised the recovery criteria, causing active cases to plummet (CTV News 2020b) and hence the gap in the data

Our simulation initializes the value of *E*, *I*, and *R* to 0. *S* is obtained by subtracting the sum of *E*, *I*, and *R* from the total population *N*. We then perform updates based on Eqs. 1–4. For all our experiments, we adopted mean latent period *C* to be 5 and mean infectious period *D* to be 14 suggested by WHO (2020b). The transmission rate $\beta _{CAN}$ is obtained by fitting Flight-SEIR to country-specific COVID-19 dataset published by the government of Canada (2020b, a). We use $R0_{CAN}$ to refer to the population reproduction number of Canada. Note that, between April 2–30, a computer error resulted in 1317 missing positive COVID-19 cases in Quebec (CTV News 2020a), causing the gap in Figs. 7, 8, 9 and 10. On July 17, Quebec revised the recovery criteria, causing active cases to plummet (CTV News 2020b) and hence the gap in Figs. 11, 12, 14, and 15.

### Predict uncontrolled early time epidemic dynamics

In the early stage of an outbreak, the majority of confirmed cases are imported from outside populations. By monitoring flights from countries with confirmed cases, Flight-SEIR can estimate the risk of an outbreak caused by the inflow of pre-symptomatic cases and provide timely signals to the need for travel restrictions and contact tracing. In particular, Flight-SEIR can provide an alert when the size of an outbreak exceeds a given threshold. In our simulations, we consider an outbreak occurs when the percentage of infected cases in the total population exceeds 0.05%. This is in accordance with the World Health Organization(WHO)’s definition of a disease outbreak (the occurrence of disease cases in excess of normal expectancy) (WHO 2020c).

To predict early time dynamics without travel restrictions, we run Flight-SEIR on flights network generated with a scaling factor of $\tau =1$, simulating the air traffic prior to the pandemic. The population reproduction number $R0_{i}$ can be chosen based on existing research on the pathogen and can be adjusted as more information becomes available. In this paper, we consider a range of [0.3, 1.2] for $R0_{CAN}$. With the basic reproduction number $R0$, many works (Verity et al. 2020; Abdollahi et al. 2020) reasoned that its early estimation can be inaccurate due to reasons such as lack of testing capabilities. As the population reproduction number $R0_{i}$ can suffer from the same pitfall, we experiment with a wider range of parameters to include both the best and worst case scenarios. The simulation runs from January 2nd, when there is no confirmed cases, to June 1st, when the travel restrictions have taken full effects.

#### **Observation 1**

*When imported cases from outside the population are incorporated into the model, even* $R0_{i}$ $< 1$ *can lead to an outbreak*.

Figure 8 shows the early time prediction for Canada, assuming no travel restriction is imposed. As shown in the figure, more than 0.051% of the population will be infected by June 1st if $R0_{CAN}$ $\ge 0.5$. More than 0.100% of the population will contract the disease if $R0_{CAN}$ $\ge 0.8$. Moreover, the number of cases is still growing by the time the simulation ends. This is in contrast with the belief in traditional epidemiology that the disease will die out when the average number of susceptible people an infectious person infects is less than 1($R0$ $< 1$) (Barabási 2013). This seemingly contradicting result can be explained by the different assumptions made by these two models. The standard SEIR model assumes that the population is a closed system where individuals do not move between the populations. However, this is often unrealistic considering the huge amount of domestic and international flights annually. On the other hand, Flight-SEIR considers demographic dynamics between populations. As a result, even if each infectious individual infects less than one susceptible person, the disease can still persist because exposed individuals from other populations can move into the population of interest and transition to the infectious compartment.

### Assess the impact of travel restrictions

To estimate the real impact of travel restrictions, we run Flight-SEIR with real flights data and the same set of $R0_{CAN}$. By comparing the results for this experiment with those in “Predict uncontrolled early time epidemic dynamics” section, we can investigate the effect of reducing air traffic on the spread of the disease. Table 2 further quantifies the effect in terms of the difference in the infected individuals in both scenarios.

#### **Observation 2**

*Travel-related NPIs have a significant impact on the spread of the disease*.

**Table 2:** Estimated percentage of infected people with and without travel restrictions on June 1st

***R0***_***CAN***_	Without travel Res. (%)	With travel Res. (%)	Difference (%)
0.3	0.036	0.003	0.034
0.4	0.043	0.004	0.038
0.5	0.051	0.007	0.045
0.6	0.063	0.011	0.052
0.7	0.078	0.017	0.061
0.8	0.100	0.027	0.073
0.9	0.130	0.043	0.087
1.0	0.172	0.066	0.105
1.1	0.231	0.102	0.128
1.2	0.313	0.156	0.157

Figure 8 projects the spread of disease in Canada with travel restriction and Table 2 shows the results in percentage. We can see that imposing travel restrictions can reduce the percentage of infectious individuals by up to 0.157% of the total population. Moreover, the impact of travel restriction increases with $R0_{CAN}$. With $R0_{CAN}$ $=0.5$, the infected population remains stable at 0.051% without travel restriction whereas the curve peaks in late February and drops to 0.007% with travel restriction. With $R0_{CAN}$ $=1.2$, the infected population is estimated to reach 0.313% if the government had not issued the travel restrictions. In the meantime, only 0.156% of population will be infected if the measure is in place.

### Estimate the population reproduction number

Flight-SEIR offers a way to decouple external source of infection with community transmission and provides a better evaluation of the current situation, represented by $R0_{i}$ (the average number of infections caused by infectious individuals of population *i*, $I_{i}$). Flight-SEIR can also infer the initial size of infected population from the dynamic flight network whereas the standard SEIR model requires at least one exposed or infected individual to be present in the population at $t=0$.

To estimate $R0_{CAN}$, we run Flight-SEIR with real air traffic and search through a set of $R0_{CAN}$. We select the one with the least mean squared error (MSE) compared to the confirmed cases. When estimating $R0_{CAN}$, one challenge is that the data we are fitting to does not necessarily reflect the reality due to insufficient testing (Verity et al. 2020; Abdollahi et al. 2020). Flight-SEIR almost always predicts more than what was reported but it does not mean that Flight-SEIR overestimates the number of infected people. To account for this, we compute MSE only for time steps where test rate exceed certain threshold. To provide a point for comparison, we also run the standard SEIR model and fit $R0$ with the same loss function.

Figure 9 shows the estimation of $R0_{CAN}$ given by Flight-SEIR and $R0$ given by the standard SEIR model for Canada. To illustrate what happens if we start SEIR before there are any confirmed cases, we run Early Start SEIR (the dashed purple line) for which we set the initial value of *E* and *I* both to 0. We see that its prediction remains a flat line throughout the experiment. We start another SEIR model (the solid purple line) the date on which total confirmed cases in Canada exceed 100 people. We search through $R0$ range of [2.50, 3.45] and find the best fit to be 2.95. We observe that, at the beginning, the standard SEIR model underestimates the number of infectious individuals compared to the confirmed cases. However, its prediction grows exponentially and shows signs of overestimation by the end of May. For Flight-SEIR, the $R0_{CAN}$ range is set to be between 0.30 and 1.25 and the best fit is estimated to be 1.05. Flight-SEIR is predicting more infectious individuals than the confirmed cases up until late April but its curve fits almost perfectly afterwards.

#### **Observation 3**

$R0$ *predicted by the standard SEIR model, which ignores the population flow, is significantly higher than* $R0_{CAN}$ *estimated by Flight-SEIR*.

This observation is for population in Canada, but the intuition should generalize to any population that is not the source of the pandemic. The best fit $R0$ (for the standard SEIR model) is 2.95 whereas the best fit $R0_{CAN}$ (for Flight-SEIR) is 1.05. One explanation is that the standard SEIR model assumes that the population is closed and all infections come from within. As a result, it requires a high $R0$ to sustain a fast initial growth. On the other hand, Flight-SEIR acknowledges that there can be exposed individuals coming from outside the population and therefore requires a lower rate for community transmission.

#### **Observation 4**

*Flight-SEIR predicts more infected individuals than the data at the beginning of the outbreak and the difference between the two approximates the degree of under reporting*.

Flight-SEIR predicts that there were 21,722 infected individuals in Canada by the time WHO declared COVID-19 as pandemic on March 11. In the meantime, Canadian government only reported 100 confirmed cases (Government of Canada 2020a). By the time all provinces or territories in Canada declared a state of emergency or a public health emergency on March 22, Flight-SEIR estimates 24,482 people to have been infected while only 1438 cases were confirmed. The discrepancy between our model prediction and official report can be attributed to insufficient testing because as testing rate went up, the difference diminished. From March 22 to June 1st, the testing rate in Canada went from 2.618 (tests per thousand) to 44.812 (tests per thousand) (Roser et al. 2020) while the difference between the model prediction and the data shrunk from 21,622 to only 10 infected people.

Flight-SEIR can incorporate realistic estimation of starting conditions if it is available. For example, suppose we assume that in early March, 20% of infected cases are reported, we can begin the simulation in March and set the initial size of infected people to be 5 times the confirmed cases on the start date. Figure 10 shows Flight-SEIR ’s prediction for this scenario. With this additional information, Flight-SEIR predicts that there were 5788 infected individuals in Canada by the time WHO declared COVID-19 as pandemic on March 11. By the time all provinces or territories in Canada declared a state of emergency or a public health emergency on March 22, Flight-SEIR estimates 10,431 people to have been infected. When we incorporate realistic estimation of the starting condition, the degree of early phase overestimation is alleviated while the fit to later phase remains reasonable.

Figure 11 shows the fitting for different provinces and territories within Canada. In almost all cases, Flight-SEIR demonstrates a better fit than the standard SEIR model. Note that we limit the date range to be between January 2nd and June 1st because Flight-SEIR is only designed to account for travel-specific measures and does not consider later changes in $R0_{CAN}$ due other NPIs such as social distancing.

### Evaluate the risks of lifting travel restrictions

Next, we study the impact of lifting travel-related NPIs such as resuming flights and opening borders. We assume the reopen date to be August 1st and run Flight-SEIR with air traffic generated from various scaling factor $\tau$ to simulate reopening at different scales. More specifically, we model the scenarios in which we resume 25%, 50%, 75% and 100% of air traffic. We obtain $R0_{CAN}$ by fitting Flight-SEIR to confirmed cases from June 1st to August 1st, approximately 2 months before the reopen date. We assume that it remains the same for the duration of the simulation. Figure 12 shows the fitting for Canada. We did grid search in the range of [0.50, 0.80] and found the best fit to be 0.63. We can tell from the curve that the number of cases already started to drop and the disease appears to have been contained.

**Fig. 13:**
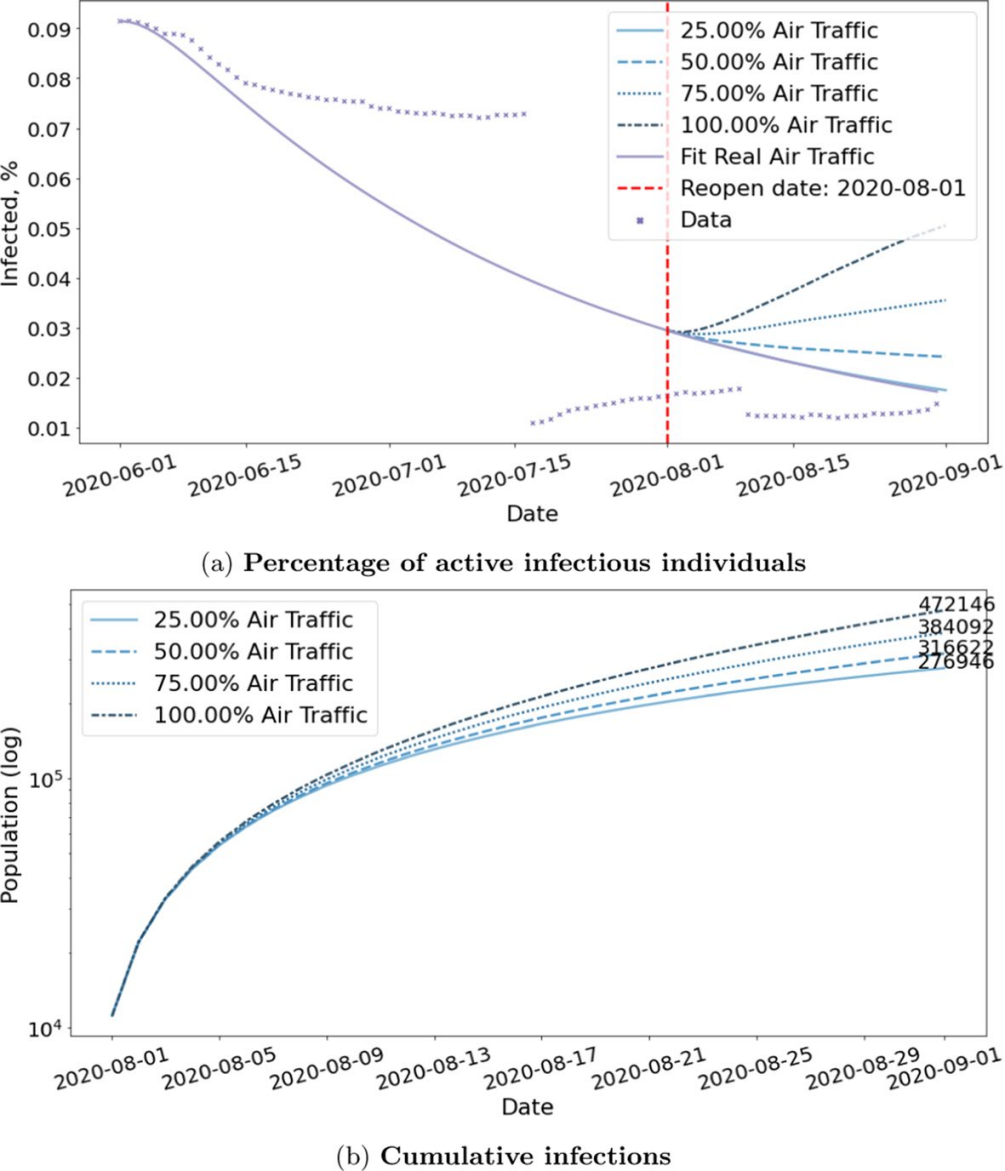
Simulation of resuming international flights in Canada. The figure shows the effect of resuming 25%, 50%, 75% and 100% air traffic between Canada and the rest of the world. The simulation starts on August 1st and continues for a month. We observe an immediate rebound when flights are increased by more than 50%

Figure 13 and Table 4 show the projections for resuming flights to and from countries all over the world. In addition, we run Flight-SEIR with real air traffic and plot its prediction as well as the confirmed cases as a baseline. Running Flight-SEIR with real air traffic and 25% air traffic produce similar curves, indicating that the real volume in August is only 1/4 of what it was before. When we resume flights for 50% or more, we immediately see a rebound in the number of infectious people. The model shows that if we resume 100% air traffic, we would have 472,146 cumulative cases by September 1st compared to only 276,946 infections if we remain at 25%.

#### **Observation 5**

*Both the number of imported cases and community transmission increase with the scale of reopening*.

**Fig. 14:**
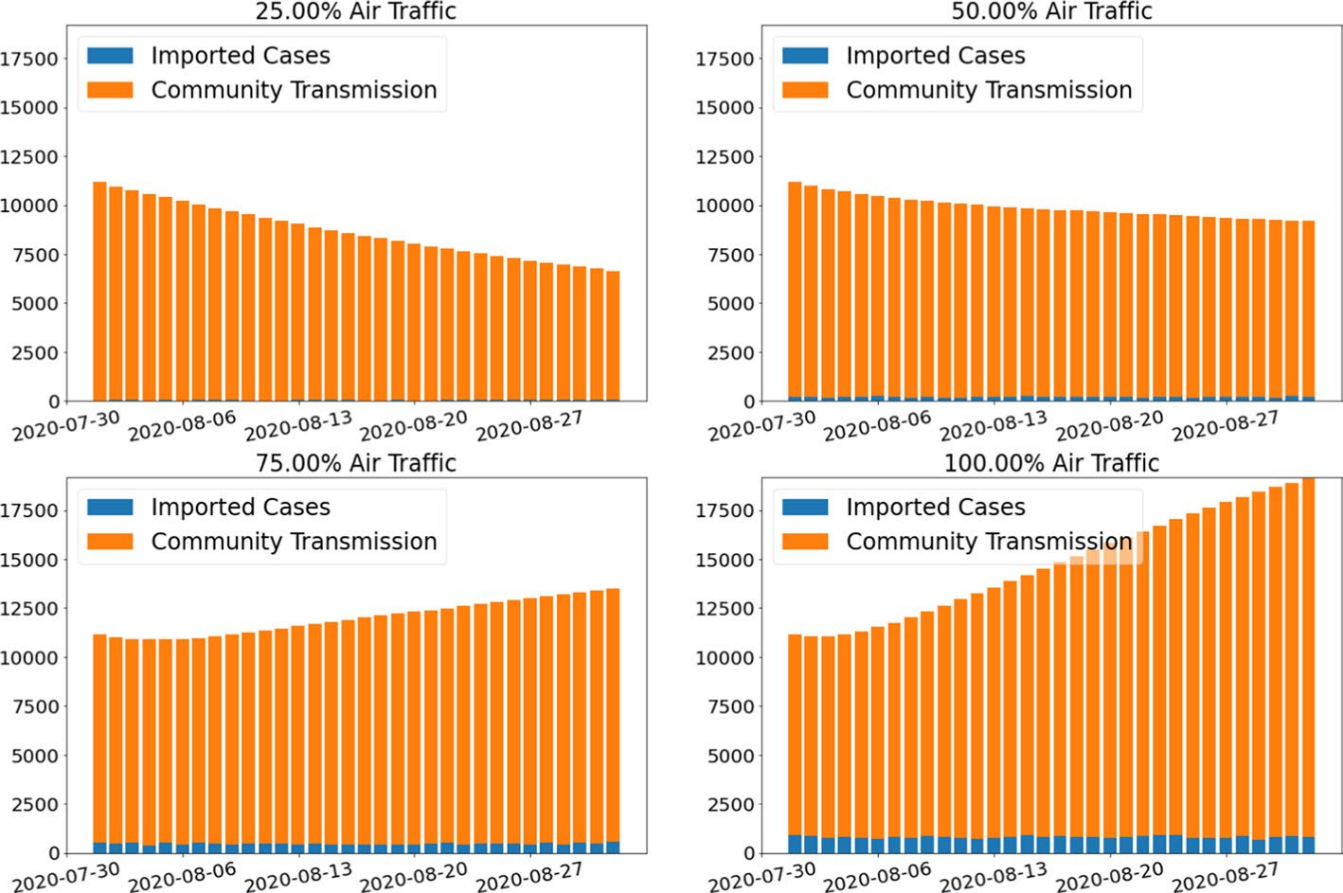
Daily active cases by source of infection. The figure shows the composition of infected population if we resume 25%, 50%, 75% and 100% of air traffic. Both the number of imported cases and community transmission increase with the scale of reopening

Figure 14 and Table 3 show the daily active cases break down by source of infection. The number of imported cases increase with the scale of reopening. Flight-SEIR predicts that by September 1st, there will be 58 imported cases per day if we only resume 25% air traffic compared to 812 per day if we resume 100% air traffic. Flight-SEIR expects there to be 6590 community transmission per day if we only resume 25% air traffic as opposed to 18,332 per day if we resume 100% air traffic. For the same scaling factor $\tau$, the ratio of imported cases to community transmissions will decrease if we lift travel restrictions for a long period of time. From August 1st to September 1st, if we resume 100% air traffic, the daily imported case will decrease from 908 to 812 whereas the daily infection caused by community transmission will increase from 10,265 to 18,332. The ratio of imported cases to community transmission decreases from 0.088 to 0.044. This may be because with the imported cases, we have a larger infected population base to infect other people and thus more community transmissions.

**Table 3:** Daily active cases by source of infection by September 1st if we resume flights between Canada and the rest of the world by 25%, 50%, 75% and 100%

Scaling factor $\tau$	Imported	Community	Total	Imported/community
0.25	58	6590	6648	0.0087
0.50	197	8993	9190	0.0219
0.75	592	12,884	13,476	0.0460
1.00	812	18,332	19,144	0.0443

#### **Observation 6**

*The effect of reopening depends on countries and we need to evaluate the risk of lifting travel restrictions on a case-by-case basis*.

**Fig. 15:**
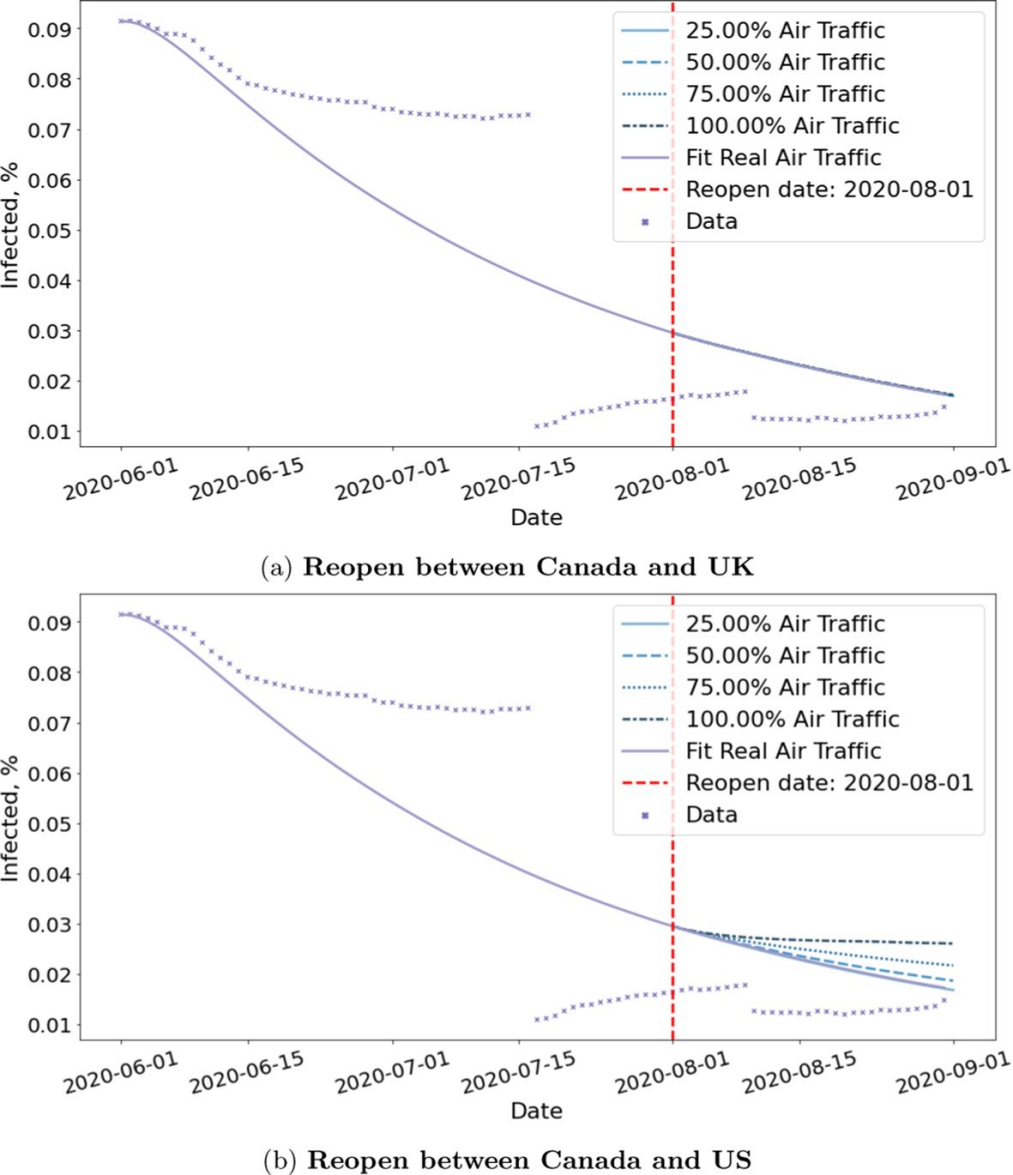
Reopening simulation with two different countries.This shows the effect of resuming 25%, 50%, 75% and 100% air traffic between Canada and UK/US. The impact of resuming flights with UK is negligible when compared to that of US

Figure 15 and Table 4 show the results for reopening with the US versus the United Kingdom(UK). We estimate that the cumulative cases will differ by 53,825 people if we resume 100% with the US as opposed to remain at current traffic. However, the cumulative cases will differ by 1264 for the UK. The impact of resuming flights with the UK is negligible compared to that of US. This can be explained by the fact that half of Canada’s international flights are coming from the US and that UK has far lower positive rate than the US.

**Table 4:** Estimated Cumulative Infections by September 1st if we resume flights between Canada and other countries by 25%, 50%, 75% and 100%

Scaling factor $\tau$	All countries	US only	UK only
0.25	276,946	273,469	275,180
0.50	316,623	283,984	275,352
0.75	384,092	302,493	275,822
1.00	472,146	327,294	276,444

#### **Observation 7**

*Resuming flights have different implications for different provinces and territories*.

**Fig. 16:**
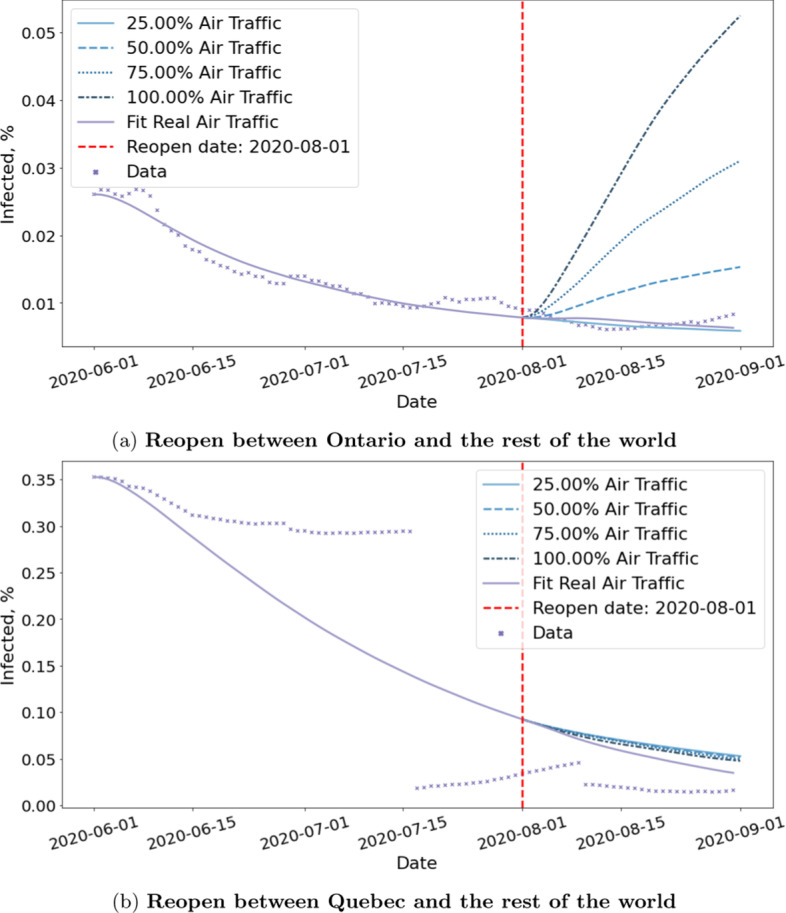
Reopening simulation for two provinces within Canada. The figure shows the effect of resuming 25%, 50%, 75% and 100% air traffic between Ontario/Quebec and the rest of the world. While lifting travel restriction is expected to have a mild impact on Quebec, we observe an immediate rebound upon reopening Ontario

Figure 16 shows the estimated risks of reopening for Ontario and Quebec. While both experiments simulate the scenario in which we resume flights to and from all countries, the impact is different for Ontario and Quebec. Ontario is estimated to have far worse rebound than Quebec. This may be due to the fact that Ontario have more international flights than Quebec in the dataset we collected. Another reason may be that Flight-SEIR consider the network flow of exposed individuals i.e. the difference between incoming and outgoing exposed. As Quebec has higher positive rate than Ontario as shown in Fig. 4, it is estimated to have much more exposed individuals leaving the province when we resume flights. Therefore, even if both provinces receive the same amount of incoming exposed people, Ontario would be at greater risks.

## Conclusion

In this work, we propose a modification to the widely used SEIR model, to derive inflow and outflow of exposed individuals from flight information. Our proposed Flight-SEIR is better suited for modelling the spread of the disease in a global pandemic such as COVID-19. The main contributions of Flight-SEIR are three-folds:

- Enables early detection of outbreaks by taking into consideration the demographic dynamics of the population.
- Provides a more accurate estimation of the parameters, in particular the population reproduction number $R0_{CAN}$, and therefore facilitates a better understanding of the disease.
- Simulates the impact of travel restriction and evaluates the implications of lifting these measures.

Even though the flight network is well recorded, access to it is still restricted. We are working towards securing access to more accurate travel records to tune our estimations. We would also like to use Flight-SEIR for modelling the spread of disease in multiple populations simultaneously. In the multi-population setting, each population or node in the flight network will have its own SEIR model and the inter-population dynamics are proxied by the flight connections. It would also be worthwhile to explore alternative distributions for latent period and infectious period (Böttcher and Antulov-Fantulin 2020). We believe that this should be the modelling used when facing a global pandemic. It is becoming even more critical with the local COVID-19 variants which although most travel restrictions are in place, are spreading between populations and becoming a major concern.

## Data Availability

The data for flight network is available from the corresponding author upon request. The code will be released upon final submission and will be available at: https://github.com/CharlotteXiaoYeDing/FlightSEIR All other datasets are publicly available: $\bullet$ Canada COVID-19 case data, test positive rate *P*: https://www.canada.ca/en/public-health/services/diseases/2019-novel-coronavirus-infection.html#a1 $\bullet$ Flights statistics: https://www150.statcan.gc.ca/t1/tbl1/en/tv.action?pid=2310000801 A $\bullet$ Population $N_i$: https://www150.statcan.gc.ca/t1/tbl1/en/tv.action?pid=1710000901 $\bullet$ Load factor *LF* and flights capacity *CAP*: https://www.transtats.bts.gov/Data_Elements.aspx?Data=5 $\bullet$ Global test positive rates: https://ourworldindata.org/coronavirus-testing

## Abbreviations

COVID-19: Coronavirus disease 2019
SEIR: Susceptible, exposed, infected, recovered
SIR: Susceptible, infected, recovered
IDVI: Infectious Disease Vulnerability Index
NPI: Non-Pharmaceutical Intervention
MSE: Mean squared error
WHO: World Health Organization
US: The United States
UK: The United Kingdom
